# Exploring the concept and structure of obstetric triage: a qualitative content analysis

**DOI:** 10.1186/s12873-020-00369-0

**Published:** 2020-09-15

**Authors:** Asieh Moudi, Mina Iravani, Mahin Najafian, Armin Zareiyan, Arash Forouzan, Mojgan Mirghafourvand

**Affiliations:** 1 Midwifery Department, Birjand University of Medical Sciences, Birjand, Iran; 2 Midwifery Department, Reproductive Health Promotion Research Center, Nursing and Midwifery School, Ahvaz Jundishapur University of Medical Sciences, Ahvaz, Iran; 3 Department of Obstetrics and Gynecology, School of Medicine, Fertility Infertility and Perinatology Research Center, Ahvaz Jundishapur University of Medical Sciences, Ahvaz, Iran; 4 Public Health Nursing Department, Nursing Faculty Aja University of Medical Sciences, Tehran, Iran; 5 Department of Emergency Medicine, Imam Khomeini General Hospital, Ahvaz Jundishapur University of Medical Sciences, Ahvaz, Iran; 6 Midwifery Department, Social Determinants of Health Research Center, Tabriz University of Medical sciences, Tabriz, Iran

**Keywords:** Obstetric triage, Concept, Structure, Qualitative, Conventional content analysis

## Abstract

**Background:**

Obstetric triage is a new idea, so the design and implementation of it requires identification of its concept and structure. The aim of this qualitative study was to explore the concept and structure of the obstetric triage in Iran.

**Methods:**

The purposive sampling was done and it continued until reaching the theoretical saturation. Thirty-seven semi-structured interviews were conducted individually and face-to-face. Interviews were audio recorded, transcribed, and analyzed using conventional content analysis.

**Results:**

Two themes, 8 main categories, and 16 subcategories emerged from the content analysis of the interviews and observations. The themes were the concept and structure of obstetric triage. The concept of obstetric triage consisted of three categories of nature, process, and philosophy of obstetric triage. The structure of obstetric triage included five categories of assessment criteria, emergency grading, determining the appropriate location for patient guidance, initiation of diagnostic and therapeutic measures, and timeframe for initial assessment and reassessment.

**Conclusion:**

Findings highlighted that obstetric triage is a process with a dual and dynamic nature. This process involves clinical decision making to prioritize the pregnant mother and her fetus based on the severity and acuity of the disease in order to allocate medical resources and care for providing appropriate treatment at the right time and place to the right patient. The results of this study could be used for the design and implementation of the obstetric triage system.

## Background

The triage concept has a historical root in military medicine and primarily focuses on mass disaster situations [1, 2]. The first triage was applied by two French military surgeons for sorting wounded soldiers in war during the years 1801–1979 [3]. In the 1950s–1960s, however, to respond to the problem of population congestion in the emergency department of hospitals, triage evolved from military to civil practice in the United States [4, 5].

Triage is widely used to refer to any decision about the allocation of limited medical resources, but Iserson et al. explained three conditions to use the term triage in emergency practice: “1- There is at least a moderate lack of health care resources. 2- A health care professional assesses the medical needs of each patient, based on a precise examination. 3- A triage officer uses a system or schedule, usually based on an algorithm or set of criteria, to determine the specific treatment or priority of treatment for each patient [1]”. Accordingly, triage involves a concise and focused evaluation and allocation of patient to a level of acuity so that the patient can safely wait for medical screening examination and treatment [6].

Triage is a risk stratification system whose extension to the obstetric service would increase the quality of patient care [7]. In 1986, obstetric triage was proposed as one of the most critical prenatal care and as a gatekeeper of the initial assessment of labor and other obstetric and non-obstetric complaints in pregnant women [6]. Since then, various studies have provided different definitions of triage. The Association of Women’s Health, Obstetric and Neonatal Nurses (AWHONN) defined obstetric triage as a concise, complete and systematic method for determining (priority for full evaluation) the disposition of the woman and her fetus [8]. This definition emphasizes that triage is the action or the methods used to assess and to prioritize patients, while some consider it as a tool of prioritization or even taking care of the place [8, 9]. On the other hand, there is no universal agreement on how patients should be triaged, nor is there a uniform law as a dynamic process [5]. Some pundits consider the concept of triage as an initial assessment by midwife or nurse with no involvement of any doctor, while others consider it as an initial assessment and evaluation [8, 10].

Obstetric triage promotes faster response to urgent situations, improves maternal and fetal care and bed utilization, avoids unnecessary admissions, reduces the waiting time and patient’s stay, increases patient satisfaction and the efficiency of departments while reducing their costs, and reduces maternal mortality [5, 9, 11, 12].

In Iran, the provision of emergency obstetric services to pregnant women is carried out at the obstetrics & gynecology emergency department of specialist hospitals or labor & delivery department of general hospitals [13]. By early 2018, emergency severity index (ESI) was used for the triage of pregnant women in general emergency wards, but obstetrics & gynecology emergency units or labor & delivery wards had no structured obstetric triage. Studies on the root causes of maternal mortality or obstetric risk management have shown that poor triage for pregnant women and defects in risk assessment are infrastructural problems of maternal mortality and obstetric adverse events [14, 15]. Therefore, in early 2018, the Ministry of Health and Medical Education prepared a draft for the obstetric triage tool and asked all its affiliated universities to submit their comments and suggestions for improvement [13]. Obstetric triage is a new idea in Iran, and it is evident that the design and implementation of an obstetric triage system requires identification of its concept and structure. Hence, this study was conducted to explore the perception of health care providers involved in obstetric triage about their concept and structure of obstetric triage. The results of this study can help improve the design and implementation of the obstetric triage system in Iran.

## Methods

### Study design and setting

This study is part of multistage mixed method research that was performed with the aim of the design, clinical validation, implementation, and determination of the feasibility of an obstetric triage system in Iran. This qualitative study involving conventional content analysis was conducted to explore the maternity care providers and policymakers’ experiences on obstetric triage during November 2018–April 2019.

The study environment was Imam Khomeini Hospital, the Deputy of Treatment, and the School of Nursing and Midwifery of Ahvaz University of Medical Sciences and the departments of midwifery, maternal health, and national triage of the Ministry of Health of Iran. The Ahvaz Imam Khomeini Hospital is a teaching tertiary referral hospital in Khuzestan province of Iran, with approximately 6700 births and 15,000 obstetric triage visits annually.

### Ethical considerations

Participants received written and oral information about the study, and written informed consent was obtained for the interview. They were free to decline participation or to withdraw at any time. The Ethics Committee of Ahvaz University of Medical Sciences approved this study (Approval ID: IR.AJUMS.REC.1397.537).

### Data collection and participants

Data were collected from in-depth, semi-structured interviews using open questions, observation, and field notes. All interviews were conducted individually and face-to-face, at a time and place that was convenient for the participant. All interviews were recorded with permission from the participants. A semi-structured preset guide was provided for the interview. The participants were asked to describe how to perform obstetric triage in the setting, and then to explain their own experiences and perceptions about obstetric triage. The focuses of interview questions were “what is your experience concerning obstetric triage?” and “what are the meaning and structure of obstetric triage in your experience?” (Additional file 1). Then, based on the participants’ responses, more open questions were asked to clarify the details of their response. Interviews were conducted between November 2018 and April 2019. Each interview lasted between 45 and 60 min and continued until reaching information saturation. The research population included 37 professional midwives, nurses, gynecologists, and specialists of emergency medicine. Participants in this study were from various occupational categories, including clinicians, hospital executive managers, faculty members, as well as provincial and national policymakers involved in the triage program. The maximum diversity in the participants was considered in terms of occupation, work experience, and education level. At first, the sampling of volunteers began with a target-based method and gradually continued through theoretical sampling. Inclusion criteria were having at least 1 year of work experience in the emergency department, and exclusion criteria were participants’ reluctance to continue collaborating.

### Data analysis

Analysis was guided by conventional (inductive) content analysis as described by Graneheim and Lunndman [16]. The analysis was carried out simultaneously with the collection of information. In this method, codes and categories were derived directly from the raw data. In this way, immediately after each interview, the entire text of it was transcribed verbatim. Each interview was read several times to identify meaning units, and then the meaning units were labeled and coded. Then meaning units and codes were classified according to conceptual and semantic similarities. Categories and sub-categories were compared together and, finally, themes were extracted from the analysis and interpretation of data.

The first author (AM) coded data initially. To obtain credibility, participants with maximum diversity were selected, sampling was done to data saturation, appropriate meaning units were selected, and data were reviewed by participants [16, 17]. The integration of data collected in the interview and field notes and checking data by foreign observers were used for dependability [18]. The research steps were fully described and were confirmed by co-authors to enhance data confirmability [18, 19]. Characteristics of the participants, methods of sampling, and data collection were described accurately to facilitate transferability [16, 18].

## Results

The findings are based on 37 interviews. Table 1 presents the participants’ characteristics. Two themes, 8 main categories, and 16 subcategories emerged from the content analysis of the interviews and observations (Table 2). The themes were the concept and structure of obstetric triage. The concept of obstetric triage consisted of three categories of nature, process, and philosophy of obstetric triage. The structure of obstetric triage also included five categories of assessment criteria, emergency grading determine the appropriate location for patient guidance, initiation of diagnostic and therapeutic measures, and timeframe for initial assessment and reassessment.

**Table 1:** Participant characteristics

Variable		Mean (SD) / frequency (number)
**Work experience**		11.1 (8.54)
**occupational categories**	Midwife	43.24 (16)
	Nurse	8.11 (3)
	Gynecologists and specialists of emergency medicine	8.11 (3)
	Assistants of gynecology and emergency medicine	18.92 (7)
	Manager	8.11 (3)
	Policymaker	13.51 (5)
**Education level**	Associate’s degree	5.40 (2)
	Bachelor’s degree	45.95 (17)
	Master’s degree	13.51 (5)
	PhD or Doctorate	16.22 (6)
	Resident in medicine	18.92 (7)

**Table 2:** Themes, main categories, and subcategories

Theme	Main categories	Subcategories
Concept of obstetric triage	Obstetric triage nature	Duality of obstetric triage Dynamic of obstetric triage
	Obstetric triage process	Clinical assessment and decision making Prioritize mothers/ fetuses Resource and medical care allocation
	Obstetric triage philosophy	Providing the right treatment to the right patient at the right time and place
Structure of obstetric triage	Assessment criteria	Maternal criteria Fetal criteria Placenta/ amniotic fluid/ cord criteria
	Emergency grading	Number and name of levels Grading criteria
	Determining the appropriate location for patient guidance	Determining the appropriate location for patient guidance
	Initiation of diagnostic and therapeutic measures	Management of low risk mothers by triage midwives Initiation of diagnostic and therapeutic measure for high risk mothers by triage midwife
	Schedule for initial assessment and reassessment	Schedule for initial assessment Schedule for reassessment

### Concept of obstetric triage

Based on the participants’ experiences and statements, obstetric triage is the dynamic and dual clinical decision-making process to prioritize patients based on the severity and acuity of the maternal and fetal condition to allocate resources and medical care to provide appropriate treatment at the right time and place to the right patient.

#### Obstetric triage nature

This category describes participant’s experiences of the dual and dynamic nature of obstetric triage.

##### Duality of obstetric triage

Regarding the subcategory of duality of obstetric triage, the participants believed that in obstetric triage, it is necessary to consider both maternal and fetal conditions.

*“According to the condition of the pregnant woman as a human and the fetus in her uterus as another human, we have two assessments, both the mother and the fetus. Any life-threatening in any of these two creates an emergency status, and w*e *have to make decisions based on that.”*(Midwife 7)

*“There are two people-there is a fetus inside the uterus- we cannot just prioritize the mother, there is a fetus as well.”*(Midwife 1)

*“Both the mother and the fetus are important. For example, umbilical cord prolapse has no danger to the mother, but her fetus is at risk of dying, and the cesarean section should be performed immediately.”*(Midwife 15)

##### Dynamicity of obstetric triage

From the participants’ point of view, triage has a dynamic nature, as at any given moment the level of illness of a person may rise from one class to another or descend to a lower class and change priorities.

*“The patient is currently in good general condition but may be in poor health in a quarter.”*(Midwife 2)

*“Triage is not only specific to the patient's arrival but must be considered throughout the patient's treatment process, as it is a dynamic process, and the patient's condition may change at any time.”*(Emergency medicine specialist 1)

*“The level of disease in some patients may rapidly change. For example, a person with severe preeclampsia may develop seizures and become a Level 1 patient.”*(Midwife 1)

#### Obstetric triage process

Participants said the triage process is a set of actions taken to achieve the primary goal of triage. These measures include clinical assessment and decision-making; prioritization of patients, and resource and medical care allocation.

##### Clinical assessment and decision-making

From the perspective of the participants, obstetric triage decision-making is a complex clinical decision based on the assessment of maternal and fetal signs and symptoms using knowledge and experience, critical thinking, and clinical judgment.

Decision-making in triage is not equivalent to a medical diagnosis, but a triage midwife should diagnose life- or organ-threatening conditions of the mother and fetus. She should be able to quickly assess the available information, identify useful clues out of them, and be able to make the right decision based on these clues, about prioritization of the patients and their place of assessment.

*“After the assessment of the patient and gathering information, I decide based on my experiences, my sixth sense, and my knowledge, that how much urgent the patient’s condition is and what I should do for her.”*(Nurse 3)

*“We assess the patient based on appearance, type of pain, chief complaint, etc., until the main symptom is identified and then start classifying based on it.”*(Midwife 2)

*“I understand the severity of the illness from the patient's symptoms, such as when a mother comes with ectopic pregnancy. Because I have seen many ectopic pregnancies, I compare her symptoms with previous cases, and I judge about her illness severity.”*(Midwife 15)

*“Diagnosis of preeclampsia is not the duty of the triage midwife; she must determine whether the patient needs to undergo resuscitation, the delivery, or the examination room at the same time or if she can wait for 10 minutes or half an hour.”*(Policy maker 1)

##### Prioritization of patients

Participants said they arrange patients to receive faster or later services and care by a physician or other health care providers.

*“When four patients come together for example, I sort them and first introduce to the physician the one who has the most urgent conditions.”*(Nurse 1)

*“When the life of a mother or fetus is at risk, we visit her earlier than those who need partial treatment or outpatient care.”*(Midwife 7)

##### Resource and medical care allocation

Participants believe that due to limited resources and health care, allocation of resources and health care based on prioritization is essential.

*“The reality is that we cannot serve multiple people at the same time, so we prioritize people based on their level of urgency and give priority to those who are in more urgency.”*(Midwife 7)

*“Because we have staff and resource constraints one way or another, we have to manage them. Emergency resources and the energy of emergency personnel should, therefore, be spent first on critically ill patients and then on better patients.”*(Policy maker 5)

#### Obstetric triage philosophy

This category justifies the existence of obstetric triage. From the participants’ point of view, the goal of creating an obstetric triage system is to ensure that the right and timely treatment is delivered to the right mother or fetus at the right time and place.

##### Providing the right treatment to the right patient at the right time and place

The obstetric triage is a way to help reduce maternal mortality by accelerating the provision of appropriate care at the right time and place to the high-risk pregnant mother. The ideal obstetric triage system should be able to accurately identify mothers or fetuses in need of emergency care, and provide conditions for quick access to medical diagnostic procedures by guiding them in the right direction and at the right time.

*“The purpose of hospital triage is to send the patient to the right place at the right time to get the right service.”*(Emergency medicine resident 1)

*“Delay in receiving appropriate care is one of the causes of maternal mortality. Thus, the triage should be able to distinguish a mother who only needs primary treatment such as serum therapy from those with emergency conditions and to direct the patient to fast track unit or waiting room and, cases that are critically ill should be transferred to the emergency room to receive faster and more appropriate treatment.”*(Policy maker 5)

*“They must have a purpose; the right decision for the right patient at the right time.”*(Midwife 5)

### Structure of obstetric triage

Participants believe that the obstetric triage structure is based on a comprehensive triage model and includes assessment criteria, emergency grading, determining the route of treatment for the patient, initiating diagnostic and therapeutic measures, and determining the timeframe for initial assessment and reassessment.

#### Assessment criteria

Participants described assessment criteria in three subcategories of maternal, fetal, and placenta/ amniotic fluid/cord.

##### Maternal criteria

Maternal criteria including gestational age, obstetric emergencies, history (obstetric, medical and pharmaceutical),chief complaints, clinical (subjective and objective) signs and symptoms, vital signs, physical examination, Para-clinic, patient inclusion, general condition, and level of consciousness.

*“The bottom line is that we give a comprehensive service to the patient. We should not ignore anything in the assessment; we should consider the mother and the fetus promptly.”*(Policy maker 4)

*“First, we need to determine whether the pregnancy is the first half of pregnancy or the second half of pregnancy. I think in the second half of pregnancy the issues, which threaten the life of the mother and a viable baby, are even more important. Emergency issues such as bleeding after an abortion or abdominal pain due to a ruptured ectopic pregnancy are important in the first half of pregnancy.”*(Gynecologist 1)

*“In obstetric triage, we assess vital signs, gestational age, co- morbidities, signs and symptoms (such as bleeding, amniotic fluid leakage, and decreased fetal movement), physical examination (such as pelvic examination), and Para-clinic tests (such as NST and ultrasound).”*(Gynecologist resident 2)

*“First, it is important to look at the appearance of the patient, for example, how the patient arrives (with his foot, wheelchair, or stretcher), or how her general condition is (ill, toxic), and her level of consciousness (alert, oriented, and unconscious)? Then the chief complaint and symptoms of the patient should be considered? Next, we need to conduct a series of physical examinations, such as checking vital signs.”*(Midwife 8)

*“We need to take a history from the patient about obstetric and gynecological conditions, medical and surgical history, and the reason of referral.”*(Midwife 5)

##### Fetal criteria

Fetal criteria included fetal heart rate, fetal movement, and diagnostic tests (NST, CST, or BPP).

*“Fetal heart rate and fetal movement in patients with higher gestational age should be controlled and decision should be made based on the fetal condition (live or dead / reduced or no fetal movement).”*(Midwife 1)

*“In our opinion, a mother with a smooth NST (without a beat to beat), is an obstetric urgency and her triage level is one, because the fetus may die in any minute.”*(Policy maker 4)

##### Placenta/ amniotic fluid/ cord criteria

The placenta, amniotic fluid, and cord were considered as assessment criteria within the structure of obstetric triage. Problems like placenta previa, placental abruption, and placenta retention or adhesion (accreta, increta, percreta) were introduced as placenta criteria. Amniotic fluid was examined in terms of volume, outflow or leakage of amniotic fluid, and color and transparency. Cord prolapse was another criterion for judging pregnant women in triage.

"*One of the cases that we consider when evaluating a patient is the placental condition. For example, pregnant women who come with placenta accreta and bleeding are placed in level one and very urgently must be transferred to the operating room or admitted to the ward".*(Midwife 9).

*“You do not see placenta retention anywhere except obstetric emergency, general emergency scales cannot be used for this patient, so you must include placenta among assessment criteria.”*(Policy maker 3)

*“We examine the clear or meconium of the amniotic fluid because thick meconium may cause fetal death, and quickly the cesarean section or vaginal birth should be done.”* (Midwife 12)

*“In patients with cord prolapse, if pulse is felt in vaginal examination, the fetus is alive and the patient is in emergency and should be immediately transferred to the operating room, but if the cord does not have a pulse, the patient is not in an emergency and must be hospitalized to give vaginal birth.”*(Midwife 11)

#### Emergency grading

This category consists of two subcategories, namely the number and name of levels, and the grading criteria.

##### Number and name of levels

Some participants said it is better for mothers/ fetuses to be categorized into 5 levels of (1) resuscitative, (2) emergent, (3) urgent, (4) less urgent, and (5) non-urgent. They believe this grading allows for more accurate evaluation and treatment that is more appropriate.

*“Dividing patients into 5 levels allows patients who need temporary admission or short-term care, such as levels 3 and 4, also receive appropriate treatment.”*(Gynecologist 1)

However, some also were of the opinion that the grading of mothers/ fetuses at three levels (resuscitative, urgent, and non-urgent) is sufficient because too many levels confuse staff and make no difference in the treatment process.

*“Classification of patients in 5 levels does not have a positive effect on the treatment, and care process, and only confuses the staff.”*(Gynecologist 2)

##### Grading criteria

Regarding the grading criteria, the gynecologists’ and midwives’ opinions differed from those of nurses. The first group grade all levels only based on the severity and acuity of the disease and the presence or absence of life or organ-threatening symptoms in the mother or fetus, because they believed that grading by the facilities needed is subjective, and various individuals may provide a different rating for a single patient. The second group reported that grading of levels 1 and 2 was conducted using the severity and acuity of illness and at other levels by the facilities required.

*“In my opinion, if levels 3 to 5 are based on the severity of the disease (as opposed to the ESI triage), assessment and prioritization will be more accurate. Because in obstetric patients, grading by facilities needed is not very precise and anyone can make different interpretation.”*(Midwifery Supervisor)

*“At levels 3, 4, and 5, where disease severity criteria are not clear, we classify mothers at these levels based on our own experience, so each of us may have different opinions about the mother.”*(Midwifery 16)

*“It is better to evaluate all levels based on the severity of the disease.”* (Gynecologist 1)

*“We first evaluate the patient based on the severity and acuity of the disease, then on the number of facilities. For example, if the patient has abdominal pain, we assess how ill she is and then estimate how many facilities (such as ultrasound and examination) she needs.”*(Nurse 2)

#### Determining the appropriate location for patient guidance

Participants stated that the triage midwives introduced a suitable place to provide proper care to the mother/fetus after assessment and categorization. Midwives entered Level 1 and Level 2 mothers to the treatment room immediately, Level 3 mothers to the fast track units or waiting room, and Level 4 and Level 5 mothers to specialized or general clinics. Sometimes mothers/fetuses may need to be referred to more equipped centers.

*“Sometimes, in-hospital visits, we see a triage nurse referring all patients to an emergency medicine specialist, but in some hospitals, the triage nurse sends some patients to the emergency medicine specialist and some to a general physician; this is the right triage. Emergency patients should be addressed to the emergency department and less urgent patients to the fast track unit and outpatient patients to the general physician.”*(Policy maker 5)

*“We send Level 1 and Level 2 mothers immediately to the delivery room or in the emergency room. For Level 3 and Level 4, it is better to have space where they can wait, Level 5 mothers are referred to either a general or specialist clinic and do not enter the emergency room.”*(Midwife 6)

*“In order to determine the mechanism and responsibilities of obstetric triage, a flowchart must be designed, and the midwife must know exactly where to send the mother.”*(Policy maker 3)

#### Initiation of diagnostic and therapeutic measures

Participants pointed out that triage midwives can manage low-risk mothers/fetuses and, in high-risk cases, they can take some diagnostic and therapeutic measures, including vaginal examination, glucometry, or pulse oximetry, and do fetal health assessment tests such as NST or request ultrasound and laboratory tests.

*“High-risk mothers should be sent to the emergency room to be examined by gynecology residents. Low-risk mothers, such as labor pain, can be managed by the midwives.”*(Gynecology resident 1)

*“For example, when a mother comes with abdominal pain and abnormal symptoms and is suspected of having an ectopic pregnancy, we do not wait for a resident to come. Initial measures such as insertion intravenous line, serum therapy, taking a sampling of the necessary tests are taken until the resident is present at the mother's bedside.”*(Midwife 3)

*“In obstetric triage, when the mother presents with a complaint of rupture of the membrane to check its severity, I do a vaginal examination, or when she complains of decreased fetal movement, I perform a none stress test.”*(Midwife 10)

#### Timeframe for initial assessment, reassessment, and evaluation

Participants stated that at any moment, the mother’s disease level might rise from one level to another or drop to a lower level, so reassessment of the mother waiting for a physician visit is necessary. They pointed out that to manage mothers faster should be specified the time threshold for midwife assessment, reassessment, and physician evaluation.

*“When triaging other mothers, we do not neglect Level 3 or 4 mothers who are waiting for a physician's visit because they may become worse (level 1 or 2) while waiting. We reassess them every 15 minutes.”*(Midwife 13)

*“Sometimes mothers who need medical counseling with other doctors (meaning doctors other than gynecologists) wait several hours. I wish that the schedule for the physician visit was defined and the physician colleagues were required to visit the mother within that schedule.”*(Midwife 4)

*“The ESI general triage has not assigned a schedule for initial and reassessment by a nurse and evaluation by a physician. In Iran, we have set a time threshold for it due to problems such as overcrowding, small numbers of personnel and resources, etc. It is also advisable to include this schedule for assessment in obstetric triage.”*(Policy maker 2)

## Discussion

This study explored Iranian maternity care providers’ and policymakers’ perceptions of obstetric triage. To the best of our knowledge, this was the first study on this subject conducted in Iran. Participants had maximum diversity in terms of occupation, work experience, and education level, which made them the best sample for answering the research question.

Although no qualitative study was found on the concept and structure of obstetric triage in the literature review, a review of existing tools showed that they are almost identical in nature, process, and philosophy. However they differ in structure (such as assessment criteria, emergency grading, etc), which may be due to variations in available resources (such as staff, place, and equipment) in various countries. (The details of the development and validation of this tool are presented in another article [20].)

A common point across the two themes was the difference between obstetric triage and general triage. This difference is due to the differences in the nature and assessment criteria of the two systems. Evidence from quantitative studies also supports the differentiation of the triage of pregnant mothers [21, 22]. All participants acknowledged the dual nature of obstetric triage. Participants explained that in obstetric triage, both fetal and pregnant mothers’ conditions should be considered in the assessment, clinical decision-making, prioritization, and allocation of resources and care. Angelini and Mahlmeister emphasized the assessment process should be done for both the mother and the fetus [23]. However, the type of general triage systems is performed only based on the conditions and symptoms of the mother’s condition, and the fetus is ignored in them. Therefore, the development of an obstetric triage system that reflects the variety of pregnant women and fetuses is essential [24].

Participants recognized that obstetric triage has a dynamic nature. Because as the patient’s condition progresses, her triage level, priority, and the intervention required could alter consequently. In agreement with our findings, Robertson-Steel [2], Gausche-Hill et al. [25], and Angelini and Mahlmeister [23] understood the change in patient status as a cause of triage dynamic, while Foley and Reisner [26] consider the shift in patient’s condition, resources, and information available during the patient response period as its reasons. Therefore, the dynamic nature is one of the characteristics of all types of triage including obstetric triage.

Participants understood the obstetric triage process as a clinical assessment and decision making to sort patients to receive care and treatment. They identified the main problem of the mother or fetus by assessing clinical signs and symptoms and then decided on her/his high-risk and life-threatening conditions using knowledge, experience, critical thinking, and clinical judgment. This decision is not equivalent to a medical diagnosis, but rather it is a rapid diagnosis of life or organ-threatening conditions of the mother and fetus and a prompt action to resolve these conditions. In accordance with our findings, Gerdtzet al. also explained triage as a decision-making process to prioritize people according to their need for medical care [27], and for this purpose identifying the chief complaint among the available symptoms and signs is crucial [28]. Numerous studies have shown the use of knowledge, experience, and intuition in triage decision-making [29–32]. Hence, like other types of triage, decision-making is an essential component of obstetric triage. Proper decision-making requires thinking and intuition gained through knowledge and experience.

Participants regarded obstetric triage as sorting or prioritizing pregnant mothers to receive care and services that allow the mothers or fetuses at high risk to receive care earlier than those with low risk. The triage is derived from the root of the French word *trier* meaning to sort. Now it is used almost exclusively in the health care context. It prioritizes high and low-risk patients to receive faster or delayed treatment [1, 33]. This definition also applies to the obstetric and general triage.

Contributors would allocate available resources and care according to the priority of pregnant mothers or fetuses because it is not possible to provide simultaneous care to all patients due to the restriction of available resources like staff, space, equipment, and the large number of patients. When the health system is not able to meet all the needs of patients with available resources, it uses a triage plan to adapt to this problem [34]. Similarly, Iserson et al. described lack of medical resources as one of the main conditions to use triage practice in this setting [1].

Participants believed philosophy of obstetric triage to ensure that the right mother/fetus goes to the right place and gets the right treatment in the shortest possible time. The participants’ understanding of the obstetric triage philosophy is similar to that of the general triage [35, 36]. Participants performed obstetric triage assessments based on maternal, fetal, and placental/amniotic fluid/cord criteria. Nevertheless, in general triage, it is based on maternal criteria only [35, 36]. This difference is due to the dual nature of obstetric triage, where maternal and fetal conditions are assessed simultaneously. On the other hand, they considered pregnancy-specific criteria such as gestational age, specific symptoms of pregnancy and childbirth, and physiological changes during pregnancy that were different from the assessment criteria of patients in general triage. Studies have also shown that the use of specific pregnancy and childbirth criteria for maternal and fetal assessment in obstetric triage is essential [21, 22].

Regarding the number of levels of obstetric triage, the participants’ opinions varied between Level 3 and Level 5. Some agreed with the five levels because they believed that this grading would result in more accurate evaluation and faster treatment of the mother or fetus. However, others disagreed with the five levels because they thought it would confuse the staff without had a positive effect on the treatment process. Various studies showed that grading patients in five levels is more effective than three levels [37, 38]. The American College of Emergency Physicians (ACEP) also supports grading patients in five levels [39]. Similarly, most of the obstetric triage systems such as OTAS and MFI also classify pregnant mothers into five levels [8, 24].

Regarding the grading criteria, participants had a different understanding. For gynecologists and midwives, the grading criterion at all levels was severity and acuity of illness, while nurses graded mother into levels 1 and 2 based on severity and acuity and into 3–5 levels based on the facilities required. The gynecologists and midwives thought that grading by the facilities needed is subjective, and various individuals may provide a different rating for a single patient. Mistry reported a similar perception among nurses in the United Arab Emirates [40]. Grading criteria in all existing obstetric triage is severity and acuity of disease [8, 24, 41].

Contributors mentioned that following initial assessment and prioritization, the mother/ fetus should be guided to the correct place of treatment. This location is determined by the level of priority of the mother/fetus. This topic is also emphasized in comprehensive models of general triage [37, 42].

Participants explained that triage midwives could manage low-risk patients and, in high-risk cases, could start some diagnostic and therapeutic measures. Paul et al. recommended the use of an obstetric triage inter-professional collaboration model between midwives and physicians. They showed that this model causes to use peoples’ professional experiences more effectively, and frees up physicians for more severe patients [43]. Similarly, Angelini et al. reported that midwives manage more than 70% of pregnant women in obstetric triage and only when a variety of obstetric complaints occur outside labor-management of physicians involved in care [44].

Due to the dynamic nature of triage, participants conducted initial assessment and reassessment for the mother/fetus. However, they pointed out that a defined schedule for them should be determined. The determination of these schedules varies in types of general triage. For example, the time to do initial assessment is only defined in the Australian triage scale (ATS) and reassessment time is considered only in Canadian triage and acuity scale (CTAS). Time to visit doctors is set almost in all types of general triage (ATS, CTAS, MTS, and ESI) [36, 45–47]. In this regard, different obstetric triages also follow the principles of general triage based on which they are designed [21, 41, 48]. Since triage aims to provide the right treatment at the right time, it seems necessary to determine the appropriate schedule for assessment and evaluation.

A limitation of this study is failure in gathering data from clinicians in non-teaching hospitals, where the process of triage of pregnant mothers differs from teaching hospitals.

## Conclusion

Findings highlighted that obstetric triage is a process with a dual and dynamic nature. This process involves clinical decision making to prioritize the pregnant mother and her fetus based on the severity and acuity of the disease to allocate medical resources and care to provide appropriate treatment at the right time and place to the right patient. The implementation of this process depends on a systematic and dependent interaction of a set of elements defined in the obstetric triage structure.

## Supplementary information

**Additional file 1.** Interview Guide

## Data Availability

All relevant data are given within the manuscript and the supplementary files.

## Abbreviations

AWHONN: Association of Women’s Health, Obstetric and Neonatal Nurses
ESI: Emergency Severity Index
ACEP: American College of Emergency Physicians
ATS: Australian Triage Scale
CTAS: Canadian Triage and Acuity Scale
MTS: Manchester Triage System
