# Supplementary material for: Exploring the concept and structure of obstetric triage: a qualitative content analysis
Source: BMC Emerg Med. 2020 Sep 15;20:74. doi: 10.1186/s12873-020-00369-0 (PMC7493847; doi:10.1186/s12873-020-00369-0)
Supplement: Supplementary file 1 — Additional file 1. Interview Guide [file 12873_2020_369_MOESM1_ESM.doc]

**Interview Guide**

**Introduction**

Thank you for accepting to be interviewed by us. The study we are undertaking is to understand more about the process of obstetric triage. I will be asking you several questions which are relevant to the study. You may respond to these queries in any way you feel comfortable. It is perfectly fine if you do not want to respond. At any point during the interview, if you are not clear about any questions, you are free to clarify the same with us and ask us to explain further. The information obtained during the interview will be kept confidential and will be shared only with the research team. We would like to audio record the interview in order to ensure that we do not miss out any salient issues. The recordings will be kept confidential. Your identity will be protected and your interview will also be labeled in codes. Is it OK with you that we audio record the interview?

**Personal Information:**

Name: __________ (optional)

Age:

Designation: Doctor/ Midwife/ Nurse

Qualification: B Sc/M Sc/PhD/MD

Months / Years of experience in maternity ward_________

What is your experience concerning obstetric triage?

What are the meaning and structure of obstetric triage in your experience?
